# Vector-mode multiplexing brings an additional approach for capacity growth in optical fibers

**DOI:** 10.1038/lsa.2018.2

**Published:** 2018-03-23

**Authors:** Alan E Willner

**Affiliations:** 1 Department of Electrical Engineering, University of Southern California, Los Angeles, CA 90089-2565, USA

The paper ‘Direct fiber vector eigenmode multiplexing transmission seeded by integrated optical vortex emitters’ by J Liu^1^ has successfully demonstrated that different data-carrying vector modes can be used to increase capacity through data-carrying, full-vectorial fiber eigenmode multiplexing communications in circular optical fibers. This work uses compact, integrated optics to achieve capacity scaling by directly exploiting orthogonal spatial vector eigenmodes.

The field of optical fiber communications has enabled the Internet transmission capacity to increase dramatically. Indeed, the capacity over an optical fiber has undergone a ‘Moore’s Law-like’ exponential increase since the 1970s. Importantly, maintaining this rate of capacity increase to meet the growth in demand has been accomplished by new technologies that emerge every few years^2^.

For the past several years, an exciting approach called space-division-multiplexing (SDM) has been proposed to be potentially used to maintain growth and multiply capacity in optical fibers^3^. SDM involves the transmission of multiple data-carrying beams simultaneously over the ‘same’ spatial medium. Two popular techniques use: (a) multicore fibers in which a single optical fiber acts as many independent data-carrying conduits by having multiple, small, isolated, high-index single-mode waveguiding cores^4^; (b) few-mode fibers in which a central larger-size waveguiding core can simultaneously accommodate multiple ‘orthogonal’ beams of light that each travel on an orthogonal spatial mode^5^. The second case, called mode-division-multiplexing (MDM), transmits multiple data-carrying beams, each on a different mode.

Crucial issues associated with MDM systems include: (a) efficiently launching each independent data stream into a different fiber mode using cost-effective components, such as a compact integrated optic element^6^; (b) mitigating the inter-modal power coupling during fiber propagation that produces channel crosstalk^7^. Such crosstalk can be handled by: (i) limiting the crosstalk occurring in the fiber to a relatively low level, which may require a special non-circular or non-central core fiber design^8, 9^; (ii) utilizing advanced digital signal processing techniques at the receiver to reduce crosstalk effects^10^. This second approach is based on the idea that different beams on different modes can represent different ‘antennas’, such that multiple-input-multiple-output algorithms, which have been used so effectively in radio, can be utilized to reduce crosstalk effects in MDM systems^11^.

A key issue with MDM systems is the use of an orthogonal modal basis set for the different beams. In general, orthogonality helps to efficiently multiplex the beams at the transmitter, demultiplex them at the receiver and lower inter-modal crosstalk during transmission.

This study focuses on optical fibers that are quite similar to the widely deployed conventional optical fibers. Such fibers have a circular, high-index-of-refraction core surrounded by a lower-index-of-refraction cladding that enables low-loss optical total internal reflection. The specific orthogonal spatial modes that can propagate in those fibers are the eigenvector solutions for wave propagation^12^. Typically, the number of spatial modes that can propagate in such fibers is related to the size of the waveguiding core. Small cores would support only a single mode (e.g., the deployed fibers for longer-distance transmission) and would not readily support MDM, whereas large cores of multimode fibers could support hundreds of modes but make mitigating inter-modal power coupling and crosstalk not readily manageable. Few mode fibers provide a nice size and system compromise.

Previously, various modal sets have been used to transmit different data-carrying beams in a circular-fiber-based MDM system. These include: (a) linearly polarized scalar modes that have tailored amplitude profiles to produce orthogonality^13^, and (b) forms of Laguerre–Gaussian modes that have tailored phasefront profiles to produce orthogonality, a subset of which are modes carrying orbital angular momentum^8, 14, 15^.

Another feature of an optical wave, in addition to its amplitude and phase, is its polarization. This leads to another type of ‘multimode’ system known as polarization multiplexing, which is typically limited to only two orthogonal modes and has been successfully and effectively incorporated into commercially available single-mode fiber systems^16^. However, more advanced polarization tailoring of beams can produce a set of values that are mutually orthogonal. Specifically, vector modes have their polarization profiles tailored in a unique manner, both radially and azimuthally, to produce a set of orthogonal modes that have a ‘vortex’ intensity profile (i.e., ring shaped with little power in the center)^17^. For example, the transverse magnetic 01 (TM_01_) mode in a fiber with no magnetic field in the direction of propagation is a radially polarized mode, while the transverse electric 01 (TE_01_) mode with no electric field in the direction of propagation is an azimuthally polarized mode.

Although other modal sets may be orthogonal, they may not be a ‘direct’ solution set for a circular-core waveguide. However, vector modes are true eigenvector modes of a circular-core fiber and represent a direct, complete, orthogonal set.

The paper on this issue entitled ‘Direct fiber vector eigenmode multiplexing transmission seeded by integrated optical vortex emitters’ by J Liu and co-workers^1^ has successfully and importantly demonstrated that different data-carrying vector modes can be used to increase capacity through full-vectorial fiber eigenmode multiplexing communications in optical fibers (Figure 1). The key advances include the observations that: (a) two different orthogonal vector modes (i.e., TM_01_ and TE_01_) were efficiently launched into a large-core fiber using two silicon-microring-resonator-based integrated photonic ‘antennas’ (i.e., vortex emitters) to properly tailor the beams; (b) each vortex beam was phase-modulated and carried independent data streams up to 20 Gbit s^−1^; and (c) beams that propagated through 2 km of fiber exhibited −16 dB intermodal crosstalk and were able to achieve a low bit-error-rate performance without the use of multiple-input-multiple-output digital signal processing at the receiver. This demonstration introduces a very exciting and valuable tool that uses compact, integrated optics to achieve capacity scaling by directly exploiting vector eigenmodes in circular fibers.

Although the MDM of vector beams was used in a free-space communication system^18, 19, 20^, it should be emphasized that the study by J Liu and colleagues is the first to report, to our knowledge, the use of data-carrying vector beams for a km-scale fiber-based MDM system. This is a significant and important step forward in the field of SDM communications.

In summary, the impact of using vector modes for SDM in fibers may be quite large for increasing the capacity of future optical fiber communication systems. Important issues for further investigation include the implications of using vector modes that are true fiber eigenmodes. Key questions include: (i) Are there scenarios for which intermodal fiber coupling and crosstalk could be lower for vector modes than for other modal sets?; (ii) are there scenarios for which vector modes are easier to generate and launch into fiber?; and (iii) are there specialty components that can enable more eigenmodes with low crosstalk?

## Figures and Tables

**Figure 1:**
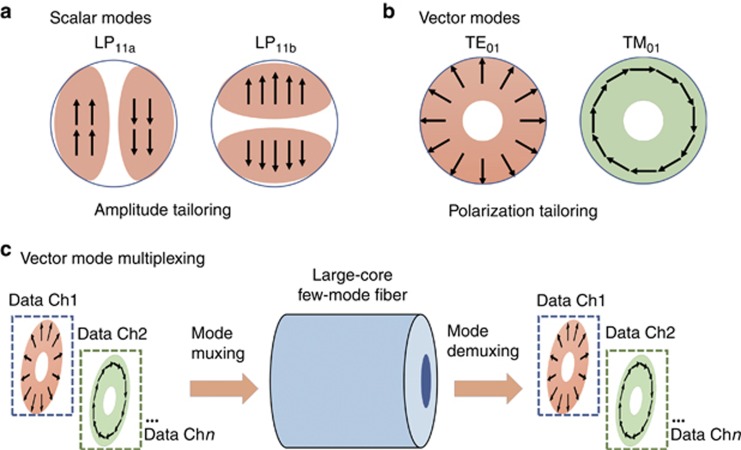
Comparison of (**a**) scalar modes (LP_11a_ and LP_11b_) and (**b**) vector modes (TE_01_ and TM_01_). Amplitude, phase and polarization tailoring of beams can be used to produce a set of mutually orthogonal modes. (**c**) Concept of fiber vector eigenmode multiplexing. Integrated photonic vortex emitters are used to tailor the beams. Ch: channel; arrows in panels a and b: polarization of electrical fields.
